# Manure application magnifies methane emission from drainage ditch sediments

**DOI:** 10.1007/s10533-026-01348-6

**Published:** 2026-06-03

**Authors:** Quinten Struik, Madison Cicha, José R. Paranaíba, Sarian Kosten, Annelies J. Veraart

**Affiliations:** Department of Ecology, Radboud Institute for Biological and Environmental Sciences, Radboud University, Nijmegen, 6525AJ The Netherlands

**Keywords:** Methane, Nitrous oxide, Carbon dioxide, Eutrophication, Fertilizer, Greenhouse gas

## Abstract

Agricultural drainage ditches have been recently identified as hotspots of methane (CH_4_), carbon dioxide (CO_2_), and nitrous oxide (N_2_O) emissions. Ditches often experience inputs of fertilizers from adjacent agricultural fields, which increases the availability of organic carbon and nutrients in ditch sediments, thereby fueling greenhouse gas (GHG) production. Here, we quantify the effects of two types of fertilizers (manure and artificial fertilizer) on GHG dynamics from agricultural drainage ditch sediments subjected to oxic and anoxic conditions. We first measured rates of potential sediment N_2_O production, CH_4_ production, and aerobic CH_4_ oxidation under different fertilizer doses. We observed that manure additions (expressed here as ammonium (NH_4_^+^) dose, referring to the resulting NH_4_^+^ concentration in a given bottle after addition) strongly stimulated CH_4_ and N_2_O production. Methane production rates increased approximately threefold as a result of manure additions, ranging from 18.4 to 61.7 µmol CH_4_ gDW^−1^ d^−1^, whereas N_2_O production rates increased approximately 16-fold, and varied from 0.1 to 1.6 µmol N_2_O gDW^−1^ d^−1^ across manure doses. Aerobic CH_4_ oxidation was also stimulated by manure addition, while at resulting NH_4_^+^ concentrations above 2 mmol L^−1^, oxidation rates declined. In contrast, artificial fertilizer caused immediate inhibition of CH_4_ and N_2_O production and aerobic CH_4_ oxidation, even at the lowest NH_4_^+^ concentration tested (0.05 mmol L^−1^). Focusing on the effects of manure on GHG emissions, we observed that under anoxic conditions, sediment cores receiving high manure inputs emitted approximately 3.5 times more GHGs (in CO_2_-equivalents) than anoxic controls (no manure) and about 5.5 times more than oxic controls. Methane was the dominant driver of increased emissions at higher manure doses. As fertilizer use continues to rise globally, these results highlight the importance of implementing climate-smart water and nutrient management strategies in ditches and adjacent grasslands to mitigate climate trade-offs.

**Supplementary Information:**

The online version contains supplementary material available at https://doi.org/10.1007/s10533-026-01348-6.

## Introduction

Agricultural drainage ditches are increasingly recognized as important sources of greenhouse gas (GHG) emissions, yet remain underrepresented in GHG inventories, particularly in the case of indirect N_2_O emissions from artificial agricultural ditches (Webb et al. 2021). These ditches are heavily managed components of agricultural landscapes, primarily constructed and maintained to drain excess water from agricultural fields. As a result, they are subject to continuous inputs of fertilizers, organic matter, and other by-products of intensive land use and agricultural practices. Fertilizer-derived nitrogen (N) and phosphorus (P) enter waterbodies such as ditches via three main pathways: (i) direct application or accidental spillage, (ii) leaching into the groundwater with subsequent discharge to surface waters, and/or (iii) surface runoff during rainfall events (Opoku et al. 2024). Each of these actions can lead to water quality problems, such as eutrophication, oxygen (O_2_) depletion, and an increase in GHG emissions, especially methane (CH_4_) (Aben et al. 2022). The high nutrient inflow drives microbial processes that produce GHGs, such as CH_4_, carbon dioxide (CO_2_), and nitrous oxide (N_2_O). Globally, ditches are estimated to account for between 0.2 and 3% of anthropogenic CH_4_ emissions (Peacock et al. 2021), and if included in global CO_2_ and N_2_O budgets, ditch emissions would lead to increases of between 0.6–1 and 3–9% in global CO_2_ and N_2_O emissions, respectively (Silverthorn et al. 2025). Additionally, in countries such as the Netherlands with extensive ditch networks, ditch CH_4_ emissions have been shown to represent between 7 and 16% of national anthropogenic CH_4_ emissions (Koschorreck et al. 2020; Peacock et al. 2021).

In the past 50 years, global fertilizer (artificial and manure) use has increased N and P application by eightfold and threefold, respectively, highlighting the intensification of agriculture to meet global demands (Lu and Tian 2017; FAO 2019), despite growing awareness of the environmental impacts. In countries with intensive agricultural activity and considerable livestock densities, such as the Netherlands, the absolute levels of N and P surpluses are substantial. In 2022, Dutch agricultural land averaged N and P surpluses of 171 and 16 kg ha^−1^ y^−1^, respectively (Wageningen University and Research 2024), while European N and P surpluses averaged 40 and 0.8 kg ha^−1^ y^−1^, respectively (EC 2021). Additionally, the use of manure increased across the country, making it responsible for 2/3rds of total national fertilizer usage (Wageningen University and Research 2024). Notably, during years of intense drought, which are expected to become more frequent with climate change (Gu et al. 2020; van der Wiel et al. 2023), fertilizer use may increase even further, to compensate for reduced crop nutrient uptake and declining yields (CLO 2025).

Although artificial fertilizer and manure are both applied to supply N, they differ fundamentally in chemical composition and biogeochemical effects. Artificial fertilizers primarily provide inorganic N, whereas manure supplies inorganic N and organic carbon, organic N, organic P, and other reduced compounds together (e.g., urea). These compositional differences strongly influence microbial metabolism. Manure can simultaneously stimulate heterotrophic respiration, methanogenesis, and denitrification-related pathways, while artificial fertilizer predominantly alters inorganic N availability alone. As a result, manure and artificial fertilizer may exert contrasting effects on sediment GHG cycling, even when the total N input is comparable (Niu et al. 2024).

Methane production in drainage ditch sediments primarily occurs through methanogenesis under anoxic conditions, particularly in nutrient-rich environments where alternative electron acceptors are depleted in sediments, such as those found in agricultural ditches (Segers 1998). Eutrophication enhances biomass production and organic matter accumulation, providing substrates that fuel methanogenesis. In agricultural ditches, nutrient inputs associated with fertilizer application have been identified as the primary drivers of GHG production and emissions, with NH_4_^+^ emerging as a key regulating variable in these systems (Wu et al. 2023). Elevated NH_4_^+^ concentrations are characteristic of fertilizer-enriched ditches and are strongly associated with increased CH_4_, CO_2_, and N_2_O concentrations and emissions, reflecting its central role in stimulating microbial metabolism and shaping redox conditions.

Alternatively, high NH_4_^+^ concentrations may exert inhibitory effects on methanogenic archaea due to ammonia toxicity, indicating that fertilizer-derived NH_4_^+^ inputs can both stimulate and suppress CH_4_ production depending on concentration, substrate availability, and microbial community responses (Angelidaki and Ahring 1993; Jiang et al. 2019). Methane produced in sediments can be partially consumed by methanotrophic microorganisms, which oxidize CH_4_ using O_2_ or alternative electron acceptors such as nitrate (NO_3_^−^) or sulfate (Segers 1998; Knittel and Boetius 2009). While eutrophication often reduces O_2_ availability and may therefore constrain CH_4_ oxidation, moderate nutrient enrichment, (including elevated NH_4_^+^ concentrations) has been shown to stimulate aerobic methanotrophy under specific conditions (van Kruistum et al. 2018; Nijman et al. 2021). However, excessive NH_4_^+^ concentrations may also inhibit CH_4_ oxidation through competitive or toxic effects on methanotrophs (Bosse et al. 1993; Van Der Nat et al. 1997). Whether NH_4_^+^-driven stimulation of CH_4_ oxidation can offset enhanced CH_4_ production and thereby reduce net emissions from agricultural drainage ditches remains uncertain.

Nitrous oxide represents an additional potent GHG (GWP of 273 CO_2_-equivalents, considering a 100-year timescale; (Calvin et al. 2023)) associated with fertilizer use. Around 60% of global N_2_O emissions originate from agriculture, primarily through microbial nitrification, nitrifier-denitrification, and incomplete denitrification processes, often enhanced by fertilizer application (Wrage-Mönnig et al. 2018; Roothans et al. 2024). Although the N_2_O yield of denitrification in aquatic systems is typically lower than in soils due to more complete reduction to N_2_ (Beaulieu et al. 2011), eutrophic aquatic systems can still constitute significant N_2_O sources because of high N inputs, sustained anoxia, and extensive land–water interfaces (van de Leemput et al. 2011; Aben et al. 2022). However, the availability of organic carbon strongly influences these pathways (Wrage-Mönnig et al. 2018), suggesting that manure and artificial fertilizer may differ substantially in their capacity to stimulate N_2_O production in ditch sediments.

Oxygen availability is a key regulator of all GHG-related processes in drainage ditches. While overlying water in ditches may experience short-term oxygenation, sediments are generally O_2_-poor due to high organic matter content and rapid microbial respiration. Slight changes in O_2_ availability at the sediment–water interface can therefore shift the balance between methanogenesis, methanotrophy, nitrification, and denitrification, ultimately determining net GHG emissions. As a result, the impact of fertilizer inputs on GHG cycling is expected to depend not only on fertilizer type and dose (Liu and Greaver 2009), but also on sediment redox conditions.

Despite increasing recognition of drainage ditches as GHG hotspots, the combined effects of fertilizer type, fertilizer dose, and O_2_ availability on sediment GHG production, oxidation, and emissions remain poorly constrained. In particular, it is unclear how manure-derived inputs, which supply both N and organic carbon, compare to artificial fertilizer inputs in shaping microbial GHG-cycling pathways under contrasting O_2_ conditions. To address this knowledge gap, we experimentally quantified the effects of increasing N-based fertilizer concentrations, applied as manure or artificial fertilizer, on GHG dynamics in agricultural drainage ditch sediments. Using a combination of batch incubations of sediment slurry and intact sediment core incubations, we aim to assess how fertilizer input influences: (i) CH_4_ and N_2_O production potentials, (ii) aerobic CH_4_ oxidation potential, and (iii) net GHG emissions from ditch sediments under oxic and anoxic overlying water conditions. We hypothesize that (H1) CH_4_ and N_2_O production initially increase with rising N-based fertilizer doses due to enhanced microbial activity; (H2) above threshold (fertilizer) NH_4_^+^ doses, NH_4_^+^ toxicity inhibits GHG production and oxidation; and (H3) fertilizer impact on GHG fluxes depends on fertilizer type and sediment oxygenation-state.

## Methods

### Experimental design and sample collection

To disentangle the effects of fertilizer type, dose, and O_2_ availability on GHG cycling in ditch sediments, we combined batch incubations and intact sediment core incubations. Batch incubations were used to quantify potential rates of key microbial processes under controlled and contrasting oxic and anoxic conditions (H1, H2). Based on the dose–response patterns identified in the batch incubations, intact sediment core incubations were subsequently conducted to quantify net GHG emissions under more ecologically realistic redox gradients and to evaluate how fertilizer dose and oxygen availability interactively control emissions at the ecosystem scale (H3).

To parameterize the experimental fertilizer doses and to define a realistic dose–response range for the incubations, we first conducted a field screening of sediment porewater nutrient concentrations in agricultural drainage ditches. In February 2024, we assessed porewater background NH_4_^+^ concentrations of the top 10 cm of sediment in 9 agricultural ditches located in Schalkwijk, the Netherlands (52° 00′ 27″ N, 5° 09′ 25″ E), where the soil is classified as ‘clay on top of peat’ (Fig. S1). Each ditch was sampled at three locations (*n* = 22): the intersection, indicating the junction between one ditch and another via a culvert, the middle, and the end (a stagnant, closed-off section). Additionally, porewater was analyzed for NH_4_^+^, NO_3_^−^, and phosphate (PO_4_^3−^) using a Bran & Luebbe continuous flow system. Measured porewater NH_4_^+^ concentrations ranged from 187 to nearly 950 µmol L^−1^ across all sites (Table S1). One ditch, designated C-MID, exhibited among the lowest average porewater nutrient concentrations (187.4 µmol NH_4_^+^ L^−1^, 0 µmol NO_3_^−^ L^−1^, and 6.40 µmol PO_4_^3−^ L^−1^) and was selected for sediment collection for batch and core incubations, allowing incubations to start from low initial NH_4_^+^ concentrations and thereby maximizing the detectable dose–response range while minimizing background input. Ditch C-MID, which was used for all subsequent sediment collections and incubation experiments, is approximately 350 m long, 3 m wide, 1 m deep, and was last dredged in August 2022 (Paranaíba et al. 2025). The surrounding grassland is used for cattle grazing from April to October each year. We chose to express fertilizer dose in terms of NH_4_^+^ concentration because NH_4_^+^ is a key driver of microbial GHG dynamics in aquatic sediments and is a strong predictor of CH_4_ and N_2_O production in agricultural drainage systems (Wu et al. 2023).

### Batch incubations of sediment slurry

**Fig. 1:**
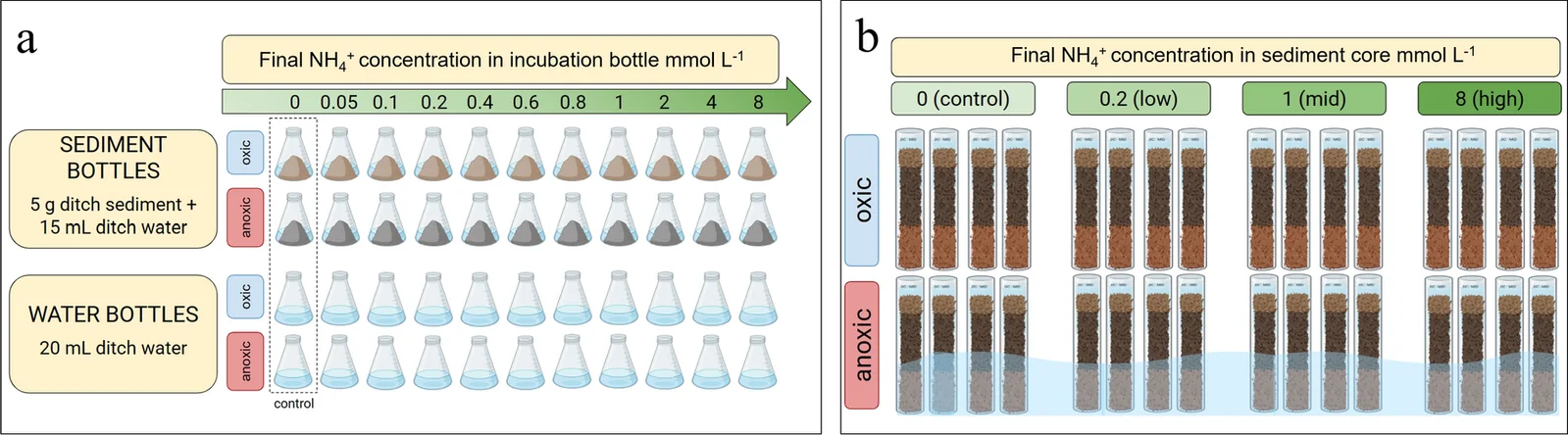
Experimental set-up of the **a** sediment slurry incubations for CH_4_ oxidation (oxic) and production (anoxic), and N_2_O production (oxic), and **b** core incubations, grouped by O_2_ treatment and manure dose (as NH_4_^+^ concentration (mmol L^−1^)). Anoxic cores were stored in anoxic water, to ensure there was no lateral O_2_ penetration into the core. Figure created using Google Slides and BioRender

We collected sediment cores (*n* = 12) in 1.8-mm-thick transparent PVC tubes (diameter 6 cm, height 120 cm) and ditch water in February and March of 2024 from site C-MID (Fig. S1). The sediment cores were collected by manually inserting the PVC tubes into the sediment until reaching the peat layer, then were immediately capped and transported to the laboratory. To assess the impact of fertilizer addition on CH_4_ oxidation and CH_4_ and N_2_O production in ditch sediment, we added 10 exponentially increasing doses (expressed as the resulting NH_4_^+^ concentrations in the incubation bottles) of fertilizer via two rounds of batch incubations (manure and artificial fertilizer; Fig. 1a), based on the NH_4_^+^ concentration found in the initial pore water samples (Tables S1 and S2). Manure was added in the first incubation round using a dilution range of extracted water from freshly collected cattle manure with a standard Rhizon type MOM sampler (Rhizosphere®). We deliberately used the water extracted from the manure rather than whole manure to represent the most likely pathway by which manure-derived nutrients and carbon reach drainage ditches. In agricultural landscapes, dissolved constituents of manure are often transported into surface waters via runoff, leaching, and tile drainage. The use of manure-water therefore most closely reflects the fraction of manure that enters aquatic systems. The extracted water was analyzed for NH_4_^+^, NO_3_^−^, and PO_4_^3−^ concentrations using a Bran and Luebbe continuous flow analyzer. Additionally, we analyzed the manure-water extract for dissolved organic carbon (DOC) and for total N concentrations using a TC-TN analyzer (Analytik Jena) and for organic carbon and C to N ratio (C:N) using a Vario Micro Cube (Elementar; Table S3). Total N concentration in the manure-water extract was 153.27 mmol L^−1^, of which 148.36 mmol L^−1^ (~ 97%) was present as NH_4_^+^. Artificial fertilizer pellets (Profijt Fertilizer NPK 12-10-18, 12% N total) used in the second incubation round were crushed into a powder and dissolved in a 20:1 stock solution (0.43 mol L^−1^ N). Sediment porewater contained low background NH_4_^+^ concentrations (Table S1) prior to amendment. Fertilizer NH_4_^+^ treatments were therefore defined based on the final NH_4_^+^ concentrations in the bottles, calculated from the experimentally added manure or artificial fertilizer.

To quantify net aerobic CH_4_ oxidation and N_2_O production under oxic conditions, we homogenized the top 2 cm of sediment from 6 cores per incubation round and added 5 g of oxic sediment + 15 mL of ditch water + their respective fertilizer dose to 120 mL gastight glass serum bottles (*n* = 10 per round). Each oxic bottle was injected at T_0_ with 1 mL of 99% pure CH_4_ gas in an oxic headspace, as in Struik et al. (2024). To quantify anoxic CH_4_ production, we homogenized the 3–6 cm layer of the remaining sediment in an anoxic glove box (O_2_ < 10 ppm) and incubated 5 g of anoxic sediment + 15 mL of anoxic ditch water (N_2_-bubbled for 1 h; dissolved O_2_ < 0.1 mg L^−1^) + fertilizer treatment in 120 mL gastight glass serum bottles (*n* = 10 per round). To correct for potential activity in the ditch water, we executed similar incubations with 20 mL of ditch water only. All bottles were closed with rubber stoppers, crimped with metal covers, and weighed at the start of the incubation period (Fig. 1a). Bottles were stored on a shaker (Edmund Buhler GmbH, KS-15), continuously shaken at 105 rpm, in a dark and fully controlled climate chamber at 18 °C (corresponding to average daily summer temperatures in the Netherlands).

We determined headspace CH_4_ and N_2_O concentrations using gas chromatography throughout the incubation periods. Aerobic CH_4_ oxidation and N_2_O production incubations were conducted over the course of 5 days, whereas anoxic CH_4_ production incubations lasted up to 12 days. The incubation duration and sampling intervals differed because they were tailored to the microbial process studied. CH_4_ oxidation was assessed over shorter intervals because it proceeds rapidly in the presence of O_2_, whereas CH_4_ production required longer incubations due to the onset of anoxic conditions and methanogenesis. Headspace CH_4_ concentrations were measured using a gas chromatograph equipped with flame ionization detection (Hewlett Packard HP 5890 Series II Gas Chromatograph, Agilent Technologies, California, USA), and headspace N_2_O concentrations were measured using a gas chromatograph/mass selective detector (Agilent 5975 C TAD Series Gas Chromatograph/Mass Selective Detector, Agilent Technologies, California, USA). Headspace samples were taken using a glass syringe (Pressure-Lok Series A-2 Syringe, Supelco, Louisiana, USA) to inject 100 µL of gas from the headspace of each bottle into the gas chromatograph at each time point (approximately once per day for production bottles and twice per day for oxidation bottles).

Oxygen was randomly measured once, nearing the end of each incubation period, using a Microx TX3 O_2_ microsensor (flat-broken needle tip, Presens, Germany) to ensure that anoxic bottles were still free of O_2_, and that oxic bottles still contained O_2_ (Table S2). At the end of each incubation period, all bottles were uncapped, and pH was measured (Table S2). We then dried them in the oven at 70 °C and obtained the dry weight of the sediment, which was used to correct for potential differences between incubated bottles. Additionally, using the original collected sediment, we dried and ground samples for organic carbon and C to N ratio analysis as described in (Vroom et al. 2020) (Table S3). Finally, anoxic CH_4_ and oxic N_2_O production rates and oxic CH_4_ oxidation rates were derived from the first linear segment of the calculated slope of the target gas concentration over time. For CH_4_ oxidation, rates were derived from the linear decrease in CH_4_ concentration and multiplied by −1 to express oxidation as a positive rate. The first linear segment typically encompassed 28 h for CH_4_ oxidation and 90 h for CH_4_ and N_2_O production in manure treatments, and 45 h for CH_4_ oxidation, 48.5 h for CH_4_ production, and 146 h for N_2_O production in artificial fertilizer treatments (Fig. S3). All rates were corrected for water content to express values per gram dry weight of sediment. Initial analyses of the incubation bottles revealed that artificial fertilizer had no measurable effect on N_2_O production and showed a late and suppressive effect in both CH_4_ production and oxidation potentials, which contrasted with the stimulatory effects observed in the manure treatments (*see Results*). Based on these findings, we focused our subsequent experiments on evaluating the impact of varying manure doses on direct GHG emissions from agricultural ditch sediments.

### Core incubations

Intact sediment core incubations were designed to quantify net GHG emissions under (compared to slurry incubations) more ecologically realistic conditions, by preserving sediment structure, redox gradients, and simultaneously occurring GHG production and oxidation pathways. Hence, to assess the effect of manure addition on net GHG emissions, we collected 32 sediment cores (diameter 6 cm, length 60 cm) in May 2024, as described earlier. The cores were left in the fridge (4 °C) for 2 weeks to settle, after which they were placed in a fully controlled climate room and incubated in the dark at 18 °C. To correct for potential differences in sediment thickness and headspace volume between sediment cores, we measured the heights of each sediment layer and the headspace. Water levels were then standardized to create a 5 cm overlying water column.

Of the 32 sediment cores, 16 were designated for oxic and 16 for anoxic incubation. Four manure treatments were established by adding manure-derived NH_4_^+^ to the sediment cores to reach calculated final core NH_4_^+^ concentrations of: 0 mmol L^−1^ (control), 0.2 mmol L^−1^ (low), 1 mmol L^−1^ (mid), and 8 mmol L^−1^ (high). These target concentrations were based only on the experimentally added manure NH_4_^+^ and did not include background porewater NH_4_^+^ or NH_4_^+^ subsequently released from the sediment. Each treatment included four replicate sediment cores (Fig. 1b). Manure extracted water was applied exclusively to the sediment surface (top layer) of each core to simulate surface manure application under field conditions. Oxic cores were flushed daily for two hours by flushing the headspace with compressed air, while anoxic cores were flushed similarly using N_2_ gas. Flushing was restricted to the headspace and did not involve bubbling or mixing of the overlying water, in order to avoid degassing of dissolved gases and physical disturbance of the water column or sediment, and to prevent CH_4_ buildup in the headspace. All cores were sealed on both ends with rubber lids and stored upright in the climate chamber. To minimize lateral O_2_ diffusion through the walls of the cores, anoxic cores were placed upright in a tub filled with anoxic water that was bubbled daily with N_2_ (Fig. 1b). The overlying water and sediment inside each core were fully isolated from the surrounding tub water. The tub water did not come into contact with the core sediment or overlying water and served solely as an external anoxic buffer. The O_2_ treatments were designed to impose contrasting and ecologically relevant O_2_ regimes in the overlying water column, allowing for disentanglement of the distinct biogeochemical processes. While O_2_ in ditch water can vary strongly over time, organic-rich sediments are generally O_2_-poor. Accordingly, oxygenation was restricted to the headspace and consequently the overlying water, whereas sediment O_2_ conditions remained largely anoxic and were mediated by diffusion. One replicate from the oxic control group was excluded due to leakage during transport, resulting in a final total of 31 cores included in the experiment.

To allow redox gradients and microbial processes to stabilize following sediment handling and manure addition, intact sediment cores were incubated for 27 days, enabling quantification of net GHG fluxes under imposed manure doses and oxic and anoxic conditions. We measured the diffusive fluxes of CO_2_, CH_4_, and N_2_O twice per week by capturing emissions with a gas-tight rubber lid connected to a greenhouse gas analyzer (Picarro G2508; closed-loop). The Picarro has a precision of 1 and 5 min, respectively, of < 10 and < 5 ppb for N_2_O, < 7 and < 5 ppb for CH_4_, < 300 and < 200 ppb for CO_2_, and a response rate of about 8 s. Prior to each emission measurement, we flushed the headspace of each core with either compressed air containing 0% CO_2_ (oxic cores) or N_2_ gas (anoxic cores) for 60 s to prevent capturing built-up gases that could skew emission measurements. Each measurement was conducted for 5 min, or until a clear linear trend in gas concentration was observed.

The water–air flux of each gas was then calculated using Eq. 1.

1 $$F= \frac{V}{A}*{\mathrm{slope}}*\frac{P*F1*F2}{R*T}$$

where *F* is the water–air gas flux (mg m^−2^ d^−1^); V is the headspace volume (m^3^), calculated as πr^2^h from the measured headspace height (*r* = 3 cm) and converted from cm^3^ prior to flux calculations; *A* is the area of the core surface (m^2^); ‘slope’ is the given gas concentration change over time (ppm s^−1^ or ppb s^−1^ for N_2_O only); *P* is the atmospheric pressure (atm); *F*1 is the molecular weight of the given gas (g mol^−1^); *F*2 is the conversion from seconds to days; *T* is the temperature in (°K); *R* is the gas constant = 8.205746 × 10^−5^ m^3^ atm mol^−1^ K^−1^.

### Statistical approach

All statistical testing was performed using R, developed by the R core team (Team RC 2013). To assess the relationship between NH_4_^+^ addition and potential anaerobic CH_4_ production, aerobic CH_4_ oxidation, and aerobic N_2_O production, we applied generalized additive models (GAMs), using the *mgcv* package. The CH_4_ and N_2_O production and CH_4_ oxidation potentials were modeled as a function of the treatment (manure and artificial fertilizer; NH_4_^+^ concentration) using a cubic regression spline, to allow for non-linear trends. Separate smooth functions were fitted for each fertilizer type (manure vs. artificial fertilizer) and were included as a parametric term to assess differences in response. Model output was assessed using the summary statistics to evaluate the significance of terms and explained variance.

We used linear mixed models (LMMs) using the *lme4* package to evaluate differences in CO_2_, CH_4_, and N_2_O emissions between the incubated cores. To meet the assumption of normality of models’ residuals, CH_4_ emission data were log_10_-transformed, while CO_2_ and N_2_O emissions were analyzed using non-transformed data. In all models, emission values were the dependent variable, and the fixed factors were fertilizer and oxygen treatments and days of incubation, while core ID was set as a random effect. Type-III ANOVAs were used to test the significance of the fixed effects. Pairwise comparisons adjusted to Tukey’s HSD as a post hoc test from the *emmeans* package were used to visualize any differences in comparisons.

To test for differences in GHG emissions expressed in CO_2_-equivalents, mean daily emissions per core were calculated across a 27-day incubation period, and were subsequently converted using their global warming potential (GWP), for CH_4_ (27) and N_2_O (273) (IPCC 2021). Generalized additive models were used to analyze the effects of manure dose and O_2_ treatment on CO_2_-equivalent emissions. Manure dose was included as a continuous predictor and O_2_ treatment as a parametric term. Gas type was included as a factor, and separate smooth terms for manure dose were fitted for each gas × treatment combination to allow for non-linear dose–response relationships.

## Results

### The effect of manure and fertilizer doses on methane and nitrous oxide production and oxidation

**Fig. 2:**
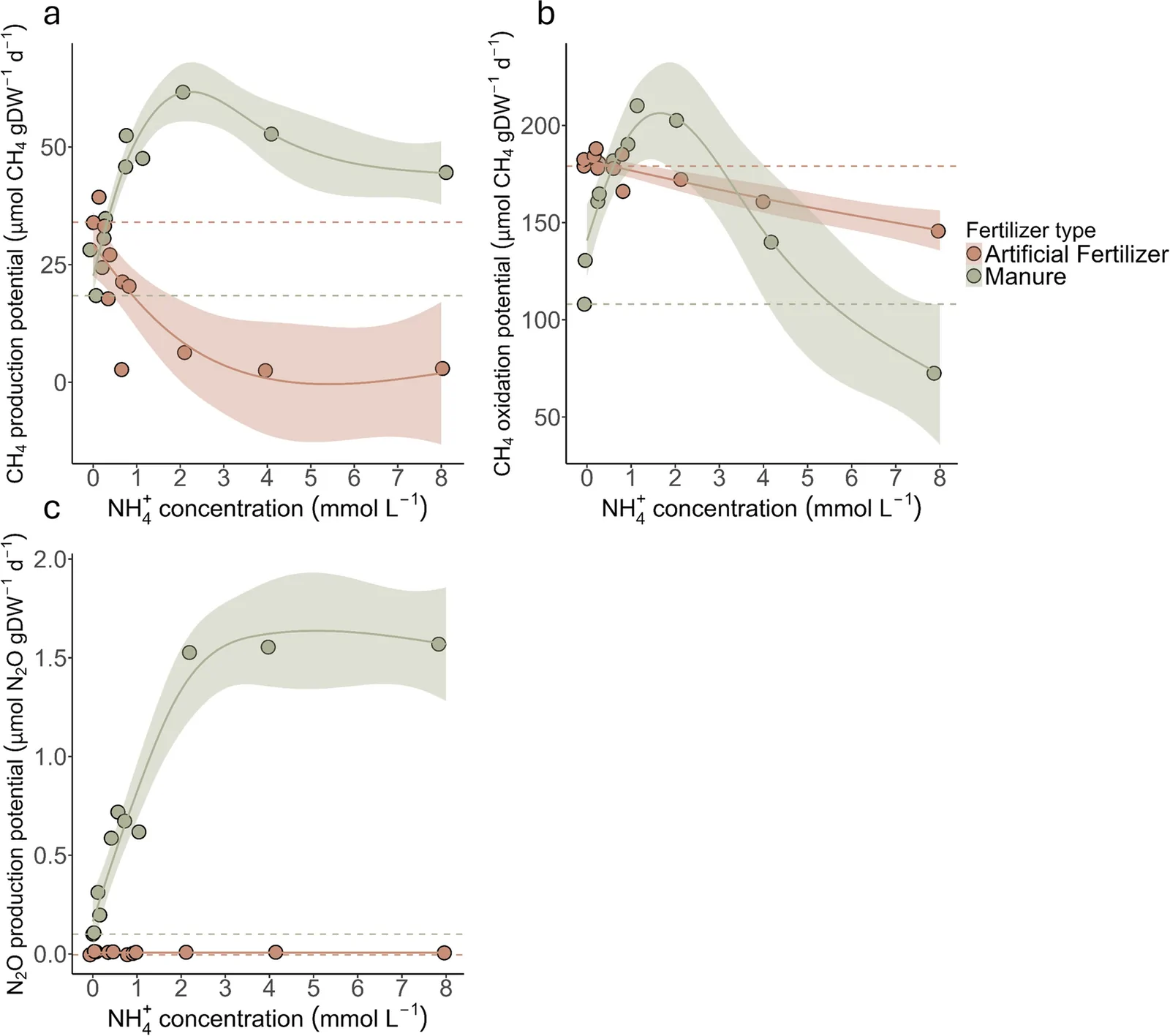
The effect of manure (green) and artificial fertilizer (brown) (expressed as resulting NH_4_^+^ concentration mmol L^−1^ in the incubation bottle) on the **a** anoxic CH_4_ production potential (µmol CH_4_ gDW^−1^ d^−1^), **b** oxic CH_4_ oxidation potential (µmol CH_4_ gDW^−1^ d^−1^), and **c** oxic N_2_O production potential (µmol N_2_O gDW^−1^ d^−1^). Dashed lines show the rate of the control bottles, and solid lines show trend lines as fitted by GAM functions, with shaded areas representing the 95% confidence interval. Batch incubation background NH_4_^+^ concentration was 0.19 mmol L^−1^

The application of artificial fertilizer and manure resulted in statistically different CH_4_ production-responses (Fig. 2a; GAM; *p* < 0.001; df = 13.6; f = 15.9). We found that sediment CH_4_ production rates decreased significantly with increasing artificial fertilizer concentrations (GAM; *p* < 0.0001; df = 9; f = 11). From 4 mmol L^−1^ NH_4_^+^ onwards, no more CH_4_ production could be detected. In contrast, increased manure doses resulted in a strong increase in CH_4_ production rates (GAM; *p* < 0.0001; df = 9; f = 10.7) and reached a maximum at 2 mmol L^−1^ NH_4_^+^, followed by a slight decrease at 4 and 8 mmol L^−1^ NH_4_^+^ concentrations (Fig. 2a). In ‘ditch water only’ treatments (without sediment), no effect of fertilizer type on CH_4_ production rates was found (Fig. S2a).

The application of artificial fertilizer and manure also resulted in significantly different CH_4_ oxidation-responses (Fig. 2b, GAM; *p* < 0.001; df = 11.1; f = 32.5). We found that the CH_4_ oxidation rates significantly decreased in response to increasing artificial fertilizer concentrations, eventually leading to CH_4_ oxidation rates below control CH_4_ oxidation rates (Fig. 2b, GAM; *p* < 0.01; df = 9; f = 6.7). Conversely, manure addition increased sediment CH_4_ oxidation rates by roughly twofold relative to the ‘no-manure’ control at a concentration of 2 mmol L^−1^ NH_4_^+^, followed by a steep decline in sediment CH_4_ oxidation rate until a concentration of 8 mmol L^−1^ NH_4_^+^, when rates were observed at nearly half those of the control (Fig. 2b). In ditch water only treatments (without sediment), no clear effect of fertilizer type on CH_4_ oxidation rates was found, and magnitudes were neglectable (Fig. S2b).

Furthermore, we found that increasing manure addition resulted in significant N_2_O production rates, reaching a plateau around 1.5 µmol N_2_O gDW^−1^ d^−1^ at 2 mmol L^−1^ NH_4_^+^ onwards (GAM; *p* < 0.0001; df = 9; f = 219.8). However, increasing artificial fertilizer addition in the same N concentration range did not result in N_2_O production (Fig. 2c).

### The effect of manure on greenhouse gas emissions under oxic and anoxic conditions

**Fig. 3:**
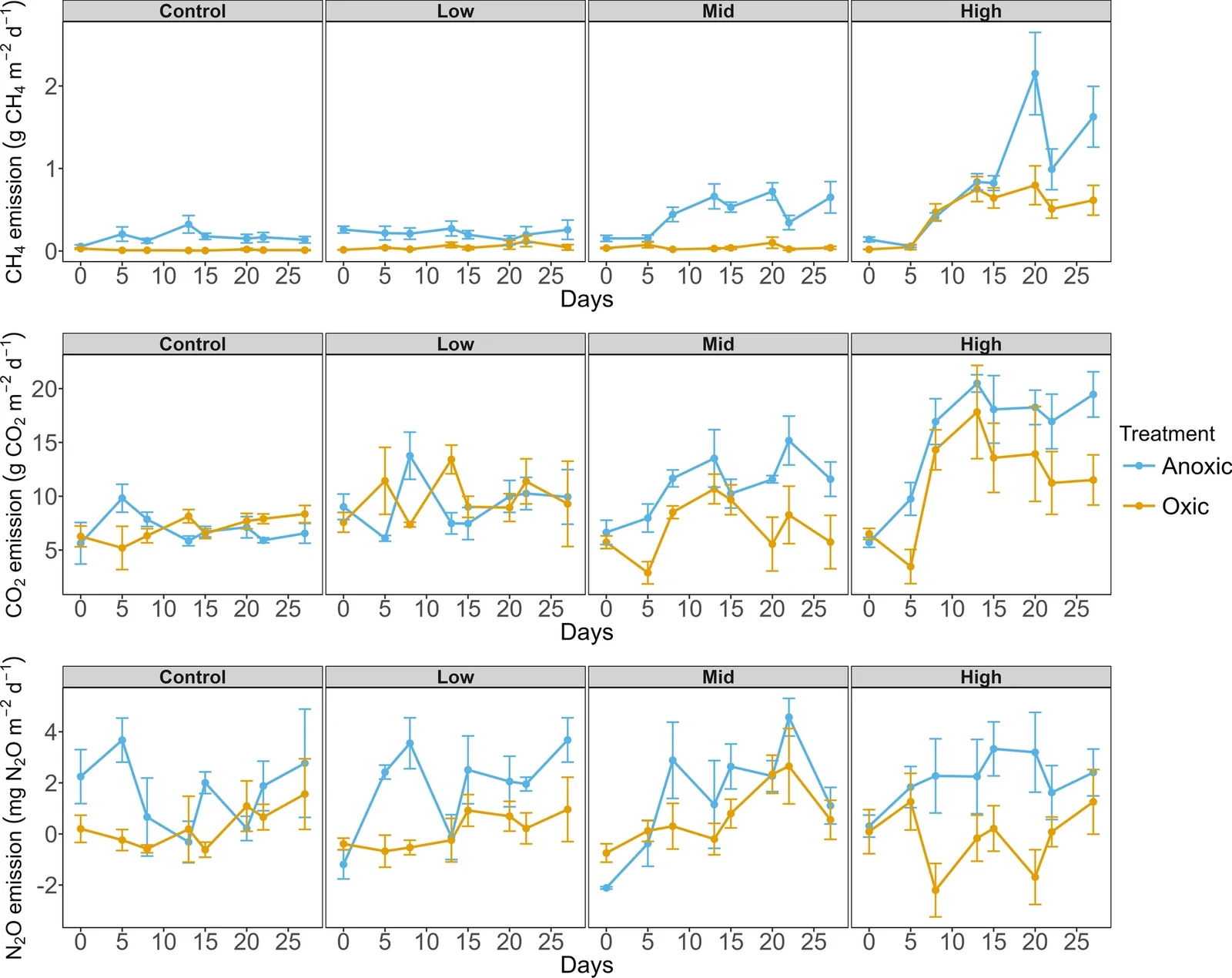
Emissions of CH_4_, CO_2_ (g m^−2^ d^−1^), and N_2_O (mg m^−2^ d^−1^) from incubated ditch sediments submitted to different doses of manure under anoxic (blue) and oxic (orange) conditions over time. Resulting sediment core manure concentrations were control (0 mmol L^−1^), low (0.2 mmol L^−1^), mid (1 mmol L^−1^), and high (8 mmol L^−1^). Dots represent mean gas fluxes, and error bars indicate ± SE

Diffusive CH_4_ emissions were significantly affected by manure doses (LMM; *p* < 0.0001; df = 20.94), O_2_ treatment of the overlying water column (LMM; *p* < 0.0001; df = 1; f = 87.95), and time (LMM; *p* < 0.0001; df = 7; f = 7.76). Significant interactions between manure dose and time (LMM; *p* < 0.0001; df = 21; f = 6.05), manure dose and O_2_ treatment (LMM; *p* < 0.01; df = 3; f = 4.6), and their three-way interaction (LMM; *p* < 0.001; df = 21; f = 2.66) indicate that the effects of manure on CH_4_ emissions changed over time and differed between oxic and anoxic conditions (Fig. 3). Post hoc comparisons (Table S4) revealed that the ‘high manure dose’ under anoxic conditions emitted significantly more CH_4_ compared to three oxic treatments, including no-manure control, low and mid manure dose treatments (Tukey HSD; *p* < 0.0001 for all comparisons; Table S4). However, there was no significant difference between oxic and anoxic high manure doses (Table S4). Mid and low manure doses under anoxic conditions also emitted significantly more CH_4_ than their oxic counterparts (Tukey HSD; *p* < 0.01; in both comparisons). Under oxic conditions, the high manure dose resulted in significantly higher CH_4_ emissions than the control, low, and mid manure doses (Tukey HSD; *p* < 0.001 for all comparisons), while low and mid oxic treatments did not differ significantly (Table S4). These results confirm a strong effect of both manure dose and O_2_ availability in the overlying water column on CH_4_ emissions, where the highest emissions occur under anoxic conditions with high and mid manure levels (Fig. 3).

Diffusive CO_2_ emissions varied significantly over time and among manure doses (Fig. 3; LMM; *p* < 0.0001; df = 3; f = 10.6). Additionally, a significant effect of O_2_ treatment on CO_2_ emissions was found (LMM; *p* < 0.05; df = 1; f = 4.7). The high manure anoxic treatment consistently emitted more CO_2_ than all oxic treatments (Table S5), except for the high manure oxic treatment. Moreover, high manure anoxic treatment emitted significantly more CO_2_ than control and low anoxic manure doses, indicating that manure addition increases CO_2_ emissions under anoxic conditions (Table S5). Additionally, there was no effect of the interaction between O_2_ treatment and manure dose on the CO_2_ emission intensity (LMM; *p* = 0.1; df = 3; f = 2.3).

Diffusive N_2_O emissions were significantly affected by O_2_ availability (LMM; *p* < 0.01; df = 1; f = 6.53) over time (LMM; *p* < 0.0001; df = 1; f = 18.7). However, no significant effect of manure dose was found (LMM; *p* = 0.3; df = 3; f = 1.25). The high manure anoxic treatment did significantly emit more N_2_O compared to control, low and high oxic manure doses (Fig. 3; Table S6), although slight increases of N_2_O emissions over time were often observed at mid and high manure levels (Fig. 3). Additionally, there was no effect of the interaction between O_2_ treatment and manure dose on the N_2_O emission intensity (LMM; *p* = 0.15; df = 3; f = 1.6).

### The effect of manure on total greenhouse gas emissions under oxic and anoxic conditions

We found a significant effect of O_2_ treatment on GHG emissions, with sediment cores under anoxic conditions emitting significantly more CO_2_-equivalents than oxic cores (GAM; *p* < 0.0001; *t* = 5.42). In addition, gas type significantly influenced emissions, with CH_4_ and CO_2_ contributing substantially more to the total emissions (expressed in CO_2_-equivalents) than N_2_O (GAM; both *p* < 0.0001).

**Fig. 4:**
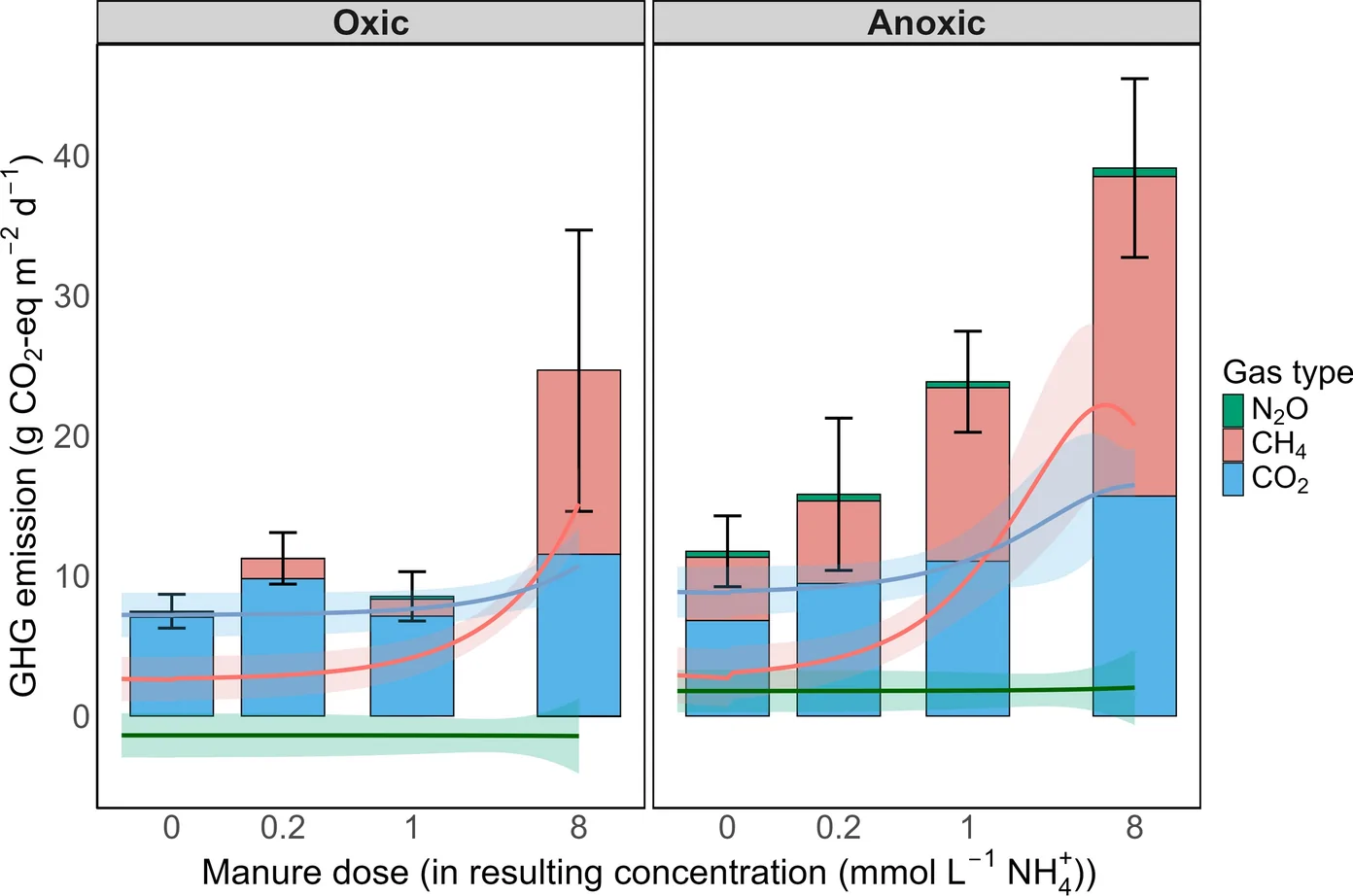
Total GHG emissions (g CO_2_-equivalents m^−2^ d^−1^) from agricultural ditch sediment cores under oxic and anoxic conditions across manure doses (mmol L^−1^). Stacked bars represent mean contributions of N_2_O (green), CH_4_ (red) and CO_2_ (blue) (± SD, *n* = 4). Solid lines show generalized additive model fits describing the non-linear relationship between manure dose and the emission of each GHG, with shaded areas indicating 95% confidence intervals

Manure addition affected the emissions of individual gases differently (see Table S7 for model parameters). We found that CH_4_ emissions increased significantly with manure dose under both oxic and anoxic conditions (GAM; oxic: *p* < 0.0001; anoxic: *p* < 0.0001) (Fig. 4). This response was particularly strong under anoxic conditions, where CH_4_ emissions increased non-linearly with manure addition. Carbon dioxide emissions also significantly increased with manure dose under both oxic (GAM; *p* = 0.049) and anoxic conditions (GAM; *p* < 0.001) (Fig. 4). In contrast, N_2_O emissions were not significantly affected by manure addition under either oxic or anoxic conditions (GAM; both *p* > 0.88).

Across treatments, CO_2_ was generally the dominant contributor to total GHG emissions. However, under high manure doses, particularly under anoxic conditions, CH_4_ emissions increased strongly and contributed nearly equally to total GHG emissions, indicating a shift towards greater CH_4_ dominance under anoxic, nutrient-enriched conditions (Fig. 4; Table S8). In addition, we found that the cores receiving the highest resulting manure dose (8 mmol L^−1^) within the anoxic treatment emitted the highest amounts of GHGs, averaging ± SD at 39.1 ± 6.4 g CO_2_-equivalents m^−2^ d^−1^ (Table S8). Anoxic cores receiving the highest manure dose emitted roughly 3.5 times more GHGs (CO_2_-equivalents) compared to anoxic no-manure controls, and approximately 5.5 times more compared to oxic no-manure controls. Moreover, under anoxic conditions, total GHG emissions increased clearly with increasing manure concentrations (Fig. 4). For the oxic treatment, manure addition resulted in comparatively smaller increases in total GHG emissions, with a marked increase only at the highest manure dose, averaging 24.6 ± 10 g CO_2_-equivalents m^−2^ d^−1^ (Fig. 4; Table S8).

## Discussion

In this study, we found that sediment CH_4_ production, oxidation, and emission were significantly affected by different manure doses. In sediment core incubations, the effects of manure were largely determined by O_2_ availability. Additionally, N_2_O production increased substantially under oxic conditions at higher manure doses, but this did not clearly translate into higher N_2_O emissions.

### Patterns of fertilizer additions on methane production, oxidation, and emission

In our bottle incubation experiments, the type of fertilizer applied strongly determined the impact on microbial CH_4_ production and oxidation. Methane production decreased with increasing artificial fertilizer additions, which aligns with previous research suggesting that high levels of NH_4_^+^ or fertilizer can inhibit methanogenic activity and change methanogenic community functioning (Yuan et al. 2018). This inhibition likely occurs due to the toxic effects of NH_4_^+^ on methanogens combined with the lack of organic carbon present in artificial fertilizer, or by competitive inhibition through stimulation of other microbial processes such as nitrification and denitrification (Yang et al. 2018). In contrast, we observed a strong increase in CH_4_ production rates with increasing manure dose. Although manure-water contained small amounts of non-ammonium N, total N measurements showed that NH_4_^+^ accounted for approximately 97% of the N in the manure extract, indicating that the contrasting microbial responses between manure and artificial fertilizer treatments cannot be attributed to unequal N inputs. Manure is a rich source of organic matter (Antil and Singh 2007), which serves as a substrate for methanogenic archaea, thereby promoting CH_4_ production (see Table S3 for TN, DOC and for org. C:N). Because manure-water was extracted using Rhizon samplers and filtered prior to analysis, measured total carbon largely represents DOC which likely provided readily available substrate fueling CH_4_ production. The extracted manure-water approach targets the primary dissolved transport pathways by which manure-derived nutrients and DOC are delivered to drainage ditches. Accordingly, our results characterize microbial responses to soluble manure inputs rather than to addition of intact manure. However, they do not encompass subsequent in situ processes whereby nutrient enrichment stimulates primary production, leading to the generation and accumulation of autochthonous particulate organic matter and its longer-term biogeochemical effects. Nevertheless, the observed maximum bottle concentration that still enhanced methanogenesis (2 mmol L^−1^ manure-derived NH_4_^+^) suggests that there might be a threshold concentration beyond which the enhancement effects of manure on methanogenesis diminish, possibly due to the inhibitory effects of higher NH_4_^+^ concentrations or self-inhibition of methanogens by product accumulation. These findings support our first hypothesis (H1) for manure, as CH_4_ production increased with higher manure dose, but also shows that very high concentrations inhibit methanogenesis. By contrast, artificial fertilizer additions immediately suppressed CH_4_ production, which does not support our first hypothesis for this fertilizer type.

We also observed a strong effect of artificial fertilizer and manure doses on potential CH_4_ oxidation rates, demonstrating a divergent response when treated with either artificial fertilizer or manure (Fig. 2). Methane oxidation rates significantly declined with increasing artificial fertilizer doses, ultimately reaching rates below those of the control sediments, suggesting a suppressive effect potentially linked to NH_4_^+^-induced or nitrite-induced inhibition of methanotrophic activity (Bosse et al. 1993; Van Der Nat et al. 1997). In contrast, manure application initially enhanced methanotrophy, doubling at concentrations of 2 mmol L^−1^ NH_4_^+^ as compared to control rates, but declining steeply at higher doses. This biphasic response implies that organic matter or nutrients in manure may transiently stimulate methanotrophs (Bodelier et al. 2000), possibly by overcoming limitations of nutrients and trace elements, either derived from manure directly or indirectly by altering heterotroph microbial composition and heterotroph-methanotroph interactions (Ho et al. 2014, 2016). The lack of initial stimulation in the artificial fertilizer treatment may in part reflect differences in baseline microbial activity or environmental conditions, as artificial fertilizer and manure incubations were conducted on sediments sampled in different months. Nevertheless, at higher doses, excessive NH_4_^+^ concentrations likely inhibit methanotrophy in both treatments (Alam and Jia 2012) (Fig. 2). Therefore, the observed biphasic pattern supports our second hypothesis (H2), suggesting that moderate manure doses stimulated aerobic CH_4_ oxidation, but high NH_4_^+^ concentrations cause inhibition. Artificial fertilizer, however, consistently suppressed CH_4_ oxidation even at the lowest concentrations, again indicating that this hypothesis only holds true for manure.

In sediment core incubations, we found that diffusive CH_4_ emissions were strongly affected by both manure dose and O_2_ availability. Emissions were highest under anoxic conditions, particularly with mid (1 mmol L^−1^) and high (8 mmol L^−1^) manure doses, which is likely the result of labile organic carbon and nutrient input strongly stimulating methanogenesis when O_2_ is limited, as observed for terrestrial soils (Kim et al. 2014). Under oxic conditions, however, the effect of manure was only apparent at the highest (8 mmol L^−1^) manure dose. This suggests that at lower manure doses under oxic conditions, net CH_4_ emissions remained limited due to a combination of processes. While CH_4_ production was modest, batch incubations showed that manure addition simultaneously stimulated aerobic CH_4_ oxidation at comparable NH_4_^+^ concentrations. In addition, aerobic heterotrophic respiration may have partially outcompeted methanogens for available carbon (Rissanen et al. 2017). Together, enhanced CH_4_ oxidation and competitive suppression of methanogenesis likely constrained net CH_4_ emissions at lower manure doses. Furthermore, the absence of significant differences between oxic and anoxic high manure doses suggests that, at high NH_4_^+^ concentrations, CH_4_ oxidation is suppressed, resulting in the highest net CH_4_ emissions, despite the oxygenation of the overlying headspace (Bosse et al. 1993; Van Der Nat et al. 1997; Jiang et al. 2019). Our observed CH_4_ emissions are likely conservative, as we did not account for ebullition. These findings show that high manure doses could substantially stimulate drainage ditch CH_4_ emissions, although the effect is highly dependent on the O_2_ state of the system.

### Nitrous oxide production and emission

During oxic batch incubations, manure application substantially increased N_2_O production rates, with rates plateauing at 2 mmol L^−1^ NH_4_^+^. In contrast, no N_2_O production was observed when artificial fertilizer was supplied. The primary N_2_O production pathway was likely microbial nitrification, where NH_4_^+^ is oxidized to NO_2_^−^ and NO_3_^−^, with N_2_O produced as a by-product (Wrage et al. 2001). Besides being a source of NH_4_^+^, the complex chemical composition of manure may have indirectly enhanced nitrification rates by stimulating microbial interactions (Laanbroek and Gerards 1991; Wrage-Mönnig et al. 2018; Li et al. 2020). Moreover, both nitrifier-denitrification and coupled nitrification–denitrification may have contributed to N_2_O production (Nielsen et al. 1996). Nitrifier-denitrification, in which nitrifiers reduce NO_2_^−^ to N_2_O, is favored under low and transient O_2_ concentrations, and especially at high NO_2_^−^ concentrations following fertilization events (Wrage-Mönnig et al. 2018). In canonical denitrification, denitrifying microorganisms use nitrification products to oxidize organic carbon, producing N_2_O as an intermediate product of NO_x_ reduction. In oxic batch incubations, denitrification may have occurred within the low-oxic zones of sediment aggregates (Wrage et al. 2001). Although the exact pathway of N_2_O production in our batch experiments remains unknown, the absence of a significant N_2_O release after artificial fertilizer application suggests that the organic fraction in manure is essential for the functioning of the N_2_O-producing microbial communities. These findings support hypothesis 1 (H1) for N_2_O in the manure treatment. However, artificial fertilizer did not enhance N_2_O production at any concentration, which refutes our expectation for that fertilizer type. This likely reflects limited stimulation of nitrification-related N_2_O-producing pathways under artificial fertilizer addition. Under oxic conditions, N_2_O can be produced via nitrification, nitrifier-denitrification, or coupled nitrification–denitrification, processes that are highly sensitive to microscale O_2_ availability and microbial activity. The manure-water contained DOC (Table S3), which may have enhanced overall microbial activity and facilitated N_2_O production within low-O_2_ microzones of sediment aggregates. In contrast, artificial fertilizer lacked a C source and therefore most likely did not stimulate N_2_O production.

In contrast to the results of our oxic batch incubations, in which increased manure dose substantially increased N_2_O production, we observed no significant effect of manure dose on N_2_O emissions across treatments during sediment core incubations. This contrast may be explained by the physical structure and redox gradients present in intact sediment cores, such as the presence of a clear anoxic zone in the sediment, which would be largely absent in homogenized, continuously shaken batch incubations. In intact partially-oxic sediment cores, the presence of an O_2_ gradient in the sediment may have facilitated complete denitrification (Parkin 1987), fully reducing NO_3_^−^, NO_2_^−^ and N_2_O to N_2_, as also confirmed by the occasionally observed negative N_2_O flux in these systems (Fig. 3). Additionally, as opposed to the batch incubations, sediment cores may not have been C and N limited, also facilitating an increase in complete denitrification rates (Firestone and Davidson 1989). Hence, in core incubations, the microbial reduction of N_2_O to N_2_ might have offset the gross production of N_2_O.

Although N_2_ production was not measured in this study, it is likely that a substantial fraction of gaseous N losses during the core incubations occurred as N_2_ rather than N_2_O. Complete denitrification commonly results in low N_2_O/N_2_ ratios, often on the order of only a few percent, although reported values are highly variable and strongly dependent on environmental conditions (Kroeze and Seitzinger 1998; Seitzinger et al. 2006). The reduction of N_2_O to N_2_ is catalyzed by nitrous oxide reductase, whose activity is known to be inhibited by O_2_ and low pH (Zumft 1997). Under the largely anoxic conditions present in the sediment cores, N_2_O-reductase activity may therefore have been favored, promoting efficient reduction of N_2_O to N_2_ and contributing to the low or even negative net N_2_O fluxes observed. Moreover, concurrent contributions from nitrification, nitrifier-denitrification, and coupled nitrification–denitrification complicate inference of N_2_O/N_2_ partitioning based on N_2_O measurements alone, suggesting that measured N_2_O fluxes represent only a minor fraction of total denitrification-derived N losses.

### Carbon dioxide and total greenhouse gas emissions

We found that the CO_2_ emissions from core incubations were determined by manure dose, suggesting that the input of organic matter significantly influences microbial activity dynamics, with CO_2_ emissions fluctuating throughout the incubation period according to the levels of manure present. Notably, the high-manure anoxic treatment consistently resulted in elevated CO_2_ emissions compared to oxic treatments, likely as the result of enhanced fermentation of DOC and nutrient input through manure addition (Hobson et al. 1974).

Under anoxic conditions, total GHG emissions (expressed as CO_2_-equivalents) were significantly higher than under oxic conditions, with the highest emissions measured in the high (8 mmol L^−1^) manure anoxic treatment. Under oxic conditions, only the highest manure dose resulted in significantly elevated GHG emissions, suggesting that O_2_ availability partially determines GHG emissions from ditches. This is likely the result of active methanogenesis under anoxic conditions, responding to increased organic carbon substrate (Aben et al. 2022). In the oxic treatment, oxygenation was initiated by flushing the headspace, after which the overlying water column may have become more oxygenated through gas exchange. The sediments likely remained anoxic beneath a thin oxic surface layer. Consequently, the contrast between oxic and anoxic treatments primarily reflects differences in O_2_ availability at the sediment–water interface rather than fully oxic versus anoxic sediment conditions. Under these circumstances, O_2_ likely regulated CH_4_ oxidation during upward transport across the interface rather than directly controlling CH_4_ production within the sediments.

When examining the absolute fluxes of the individual GHGs, we found that manure application specifically led to an increase in CH_4_ and CO_2_ emissions, particularly under anoxic conditions. While our study focuses on aquatic sediments, it is important to note that fertilizer-type effects on GHG emissions differ markedly between aquatic and terrestrial systems. In terrestrial soils, the application of artificial N-based fertilizers has been shown to result in higher total GHG emissions compared to manure fertilization (Hu et al. 2023). This contrast highlights that fertilizer-related GHG trade-offs are ecosystem-specific and that mitigation strategies targeting terrestrial emissions may have unintended consequences for connected aquatic systems.

The contribution of N_2_O to total CO_2_-equivalents remained minor due to its relatively low absolute fluxes, despite its high GWP. Our measured CH_4_, CO_2_ and N_2_O emissions are of low (Paranaíba et al. 2025) to similar (Peacock et al. 2021; Hendriks et al. 2023) magnitude as previously reported GHG emissions from agricultural drainage ditches. These findings confirm our third hypothesis (H3) as increasing concentrations of manure led to higher total GHG emissions, amplified under anoxic conditions.

Our results are based on controlled sediment core incubations and therefore do not fully capture the complexity of *in-situ* ditch conditions. In drainage ditches, GHG emissions are additionally regulated by a wide range of variables (e.g., hydrological dynamics, sediment–water exchange, environmental variability, and macrophyte abundance) (Wu et al. 2023; van der Knaap et al. 2025; Struik et al. 2026). Moreover, gas transport pathways such as ebullition and plant-mediated emissions, which can strongly influence GHG emissions, are not represented in our experimental setup (Bodmer et al. 2024). Consequently, the absolute emissions reported here should be interpreted as process-based responses rather than direct estimates of *in-situ* emissions. Nevertheless, the observed increase in CO_2_ and CH_4_ emissions with increasing manure dose, particularly under anoxic conditions, highlights the sensitivity of ditch sediments to nutrient and organic matter inputs and redox state. These conditions are commonly encountered in agricultural ditches receiving manure-derived nutrients and C inputs. Our findings therefore suggest that manure inputs can enhance the potential for GHG production in ditch sediments, with *in-situ* emissions likely depending on the duration and spatial extent of anoxic conditions and the dominant GHG emission pathway.

Currently, there is increasing global awareness of the environmental impacts of intensive livestock farming and manure management, particularly regarding nutrient leaching, eutrophication, and GHG emissions. In response, various regulatory frameworks have been implemented to mitigate these risks. Within the European Union, for instance, the application of manure is governed by the Nitrates Directive to mitigate N leaching and eutrophication (Musacchio et al. 2020). In the Netherlands, where the entire region is classified as a nitrate vulnerable zone, additional national regulations are enforced. These regulations encompass stringent limits on the application of N and P based on crop and soil type, mandatory nutrient budgeting, and requirements for manure processing or export in cases of surplus. Application is prohibited during periods of elevated leaching risk, such as wet conditions or winter, and buffer zones along water bodies are mandated to reduce runoff. Moreover, manure must be applied using low-emission techniques, such as through slurry injection, to minimize volatilization losses. However, the effectiveness of these measures in reducing the nutrient loading to ditches remains uncertain (Van Grinsven et al. 2016; Musacchio et al. 2020).

## Conclusion

We demonstrate that (1) manure dose can stimulate CH_4_ and N_2_O production in sediment batch incubations, whereas artificial fertilizer does not show this effect, and (2) elevated manure doses can increase CH_4_ and CO_2_, but not N_2_O, emissions from ditch sediments. Although in our experiments, artificial fertilizer appeared to be a more greenhouse gas-efficient option in this context, the broader environmental context often favors manure application due to its potential to enhance agricultural circularity and promote sustainable nutrient recycling (Granstedt 1991). It is important to note that these findings are specific to aquatic sediment processes and do not necessarily reflect GHG dynamics in terrestrial systems under similar fertilizer treatments. Furthermore, it is important to acknowledge that our experimental design did not consider potential indirect effects of fertilization on primary production, such as increased macrophyte growth, which could significantly alter the carbon cycle in these aquatic systems, enhancing or suppressing GHG emissions (Aben et al. 2022; Bodmer et al. 2024).

Nevertheless, our findings have broader implications for GHG mitigation in agricultural drainage ditches. Our results underscore the critical role of O_2_ limitation and nutrient loading in controlling emission dynamics. Specifically, in eutrophic ditches, periods of low or absent O_2_ availability in the overlying water may strongly amplify the stimulatory effect of manure runoff on total GHG emissions, whereas this response is likely weaker during periods with higher O_2_ availability. Improving water quality by reducing nutrient inputs and averting prolonged anoxia may therefore mitigate the stimulatory effect of manure addition on GHG emissions in agricultural drainage ditches. As the use of manure continues to rise, these results highlight the critical importance of implementing integrated climate-smart water and nutrient management strategies in the regulation of ditches and grasslands to mitigate unintended climate trade-offs.

## Supplementary Information

Below is the link to the electronic supplementary material.

Supplementary file1 (DOCX 3986 KB)

## Data Availability

The raw data collected in this study will be deposited after acceptance in DANS EASY data repository.
