# Supplementary material for: Manure application magnifies methane emission from drainage ditch sediments
Source: Biogeochemistry. 2026 Jun 3;169(4):50. doi: 10.1007/s10533-026-01348-6 (PMC13442480; doi:10.1007/s10533-026-01348-6)
Supplement: Supplementary file 1 [file 10533_2026_1348_MOESM1_ESM.docx]

**Supplementary information for:**

Manure application magnifies methane emission from drainage ditch sediments

Quinten Struik^1,*,^^, Madison Cicha^1,*^, José R. Paranaíba^1^, Sarian Kosten^1^, Annelies J. Veraart^1^

^1^ – Department of Ecology, Radboud Institute for Biological and Environmental Sciences, Radboud University, Nijmegen, The Netherlands

^*^ – Both authors contributed equally to the manuscript

^^^ – Corresponding author – quinten.struik2@ru.nl Quinten.struik2@ru.nl

Radboud Institute for Biological and Environmental Science, Radboud University, Nijmegen, 6525AJ, The Netherlands

**Supplementary material**

This supplement provides additional figures and tables that support the main findings of the manuscript. **Figure S1** presents an aerial overview of the ditch network and the sampling locations used to determine background porewater nutrient concentrations, while **Figure S2** shows the effects of manure and artificial fertilizer additions on methane production and oxidation in ditch water. **Figure S3** shows the time series plot from batch incubations for different GHGs. **Tables S1–S3** provide detailed nutrient, pH, and sediment composition data that underpin the experimental incubations. **Tables S4–S7** contain the statistical comparisons of treatment effects. Finally, **Table S8** reports average greenhouse gas emissions from sediment core incubations under different O_2_ and manure treatments. Together, these supplementary materials provide the methodological and quantitative context for the main text and substantiate the interpretation of greenhouse gas dynamics in the studied ditch system.

*Supplementary figures*

**Fig. S1** Aerial overview of the sampling location and sites used to quantify background NH_4_^+^ porewater concentrations. X’s designate different sites for sampling pore water concentration (blue = intersection; red = middle; purple = end). The yellow star (C-mid) was used as the location for the incubated sediments in this study. Light blue arrows indicate the water flow direction.

**Fig. S2** The effect of manure dose (green) and artificial fertilizer dose (brown) on the (**a**) CH_4_ production potential (µmol CH_4_ ml^-1^ d^-1^) and (**b**) CH_4_ oxidation potential (µmol CH_4_ ml^-1^ d^-1^) of the ditch water only. Dashed lines show the rate of the control bottles, and solid lines show the trend line as fitted by the GAM function, and the shaded areas represent the 95% confidence interval.

**Fig. S3** Time series of greenhouse gas concentrations during batch incubations under controlled redox conditions and increasing NH_4_^+^ doses. Panels (a–b) show CH_4_ production under anoxic conditions for manure (a) and artificial fertilizer (b). Panels (c–d) show CH_4_ oxidation under oxic conditions for manure (c) and artificial fertilizer (d). Panels (e–f) show N_2_O production under anoxic conditions for manure (e) and artificial fertilizer (f). Shaded areas indicate the time intervals over which linear slopes were calculated.

*Supplementary tables*

**Table S1:** Nutrient concentration in porewater (µmol L^-1^) from different locations within the ditch network. See Fig S1 for sampling locations.

|  | PO_4_^3-^ | NO_3_^-^ | NH_4_^+^ |
| --- | --- | --- | --- |
| A-int | 41.67 | 0.00 | 744.8 |
| A-mid | 25.63 | 0.67 | 427.9 |
| A-end | 101.76 | 0.00 | 312.7 |
| B-int | 19.07 | 0.89 | 377.5 |
| B-mid | 43.41 | 0.00 | 319.5 |
| B-end | 115.61 | 0.00 | 948.9 |
| C-int | 29.47 | 0.00 | 634.5 |
| C-mid | 6.40 | 0.00 | 187.4 |
| C-end | 31.24 | 0.00 | 480.3 |
| D-int | 37.90 | 0.00 | 207.0 |
| D-mid | 21.54 | 0.00 | 261.8 |
| D-end | 26.19 | 0.00 | 709.4 |
| E-int | 30.37 | 0.17 | 675.5 |
| E-mid | 60.47 | 0.00 | 370.1 |
| E-end | 49.60 | 0.00 | 243.2 |
| F-int | 18.58 | 0.00 | 289.7 |
| G-int | 7.91 | 1.76 | 261.0 |
| G-end | 10.98 | 0.00 | 210.1 |
| H-int | 85.65 | 0.00 | 761.9 |
| H-end | 14.76 | 2.65 | 457.5 |
| I-int | 81.30 | 0.00 | 355.8 |
| I-end | 5.80 | 0.34 | 352.0 |
| Manure | 16.34 | 0.72 | 148365 |

**Table S2:** pH and randomly measured O_2_ concentration at the end of the batch incubations according to their incubation type (oxic/anoxic), the incubated substrate (sediment/water), and fertilizer type (manure/artificial fertilizer).

| Incubation type | Sediment or water | NH_4_^+^ added (µmol L^-1^) | pH manure | pH artificial fertilizer | O_2_ concentration mg L^-1^ manure | O_2_ concentration mg L^-1^ artifical fertilizer |
| --- | --- | --- | --- | --- | --- | --- |
| Oxic | Sediment | Control | 5.44 | 5.69 | **5.9** | *NA* |
| Oxic | Sediment | 50 | 5.27 | 5.44 | **5.9** | *NA* |
| Oxic | Sediment | 100 | 5.21 | 5.42 | **5.8** | **5.79** |
| Oxic | Sediment | 200 | 5.04 | 5.34 | **5.6** | *NA* |
| Oxic | Sediment | 400 | 5.38 | 5.26 | **5.6** | *NA* |
| Oxic | Sediment | 600 | 5.19 | 5.22 | **5.5** | *NA* |
| Oxic | Sediment | 800 | 5.08 | 5.22 | **5.4** | *NA* |
| Oxic | Sediment | 1000 | 5.41 | 5.45 | **5.2** | *NA* |
| Oxic | Sediment | 2000 | 5.34 | 4.92 | **4.7** | **5.47** |
| Oxic | Sediment | 4000 | 5.62 | 5.02 | **3.7** | *NA* |
| Oxic | Sediment | 8000 | 6.18 | NA | **3** | *NA* |
| Oxic | Water | Control | 6.47 | 7.3 | *NA* | *NA* |
| Oxic | Water | 50 | 6.44 | 7.13 | *NA* | *NA* |
| Oxic | Water | 100 | 6.43 | 6.88 | *NA* | *NA* |
| Oxic | Water | 200 | 6.47 | 6.89 | *NA* | *NA* |
| Oxic | Water | 400 | 6.63 | 6.82 | *NA* | *NA* |
| Oxic | Water | 600 | 6.67 | 6.91 | *NA* | *NA* |
| Oxic | Water | 800 | 6.79 | 6.9 | *NA* | *NA* |
| Oxic | Water | 1000 | 6.78 | 6.8 | *NA* | *NA* |
| Oxic | Water | 2000 | 7.53 | 7.23 | *NA* | *NA* |
| Oxic | Water | 4000 | 7.04 | 7.35 | *NA* | *NA* |
| Oxic | Water | 8000 | 7.51 | 7.19 | *NA* | *NA* |
| Anoxic | Sediment | Control | 6.44 | 7.39 | **0** | **0** |
| Anoxic | Sediment | 50 | 6.54 | 7.04 | *NA* | *NA* |
| Anoxic | Sediment | 100 | 6.64 | 6.99 | **0** | *NA* |
| Anoxic | Sediment | 200 | 6.55 | 6.8 | *NA* | *NA* |
| Anoxic | Sediment | 400 | 6.79 | 6.58 | *NA* | **0** |
| Anoxic | Sediment | 600 | 6.73 | 6.7 | **0** | *NA* |
| Anoxic | Sediment | 800 | 6.63 | 6..46 | *NA* | **0** |
| Anoxic | Sediment | 1000 | 6.5 | 6.81 | *NA* | *NA* |
| Anoxic | Sediment | 2000 | 6.75 | 6.84 | *NA* | *NA* |
| Anoxic | Sediment | 4000 | 6.81 | 7.19 | **0** | *NA* |
| Anoxic | Sediment | 8000 | NA | 7.03 | *NA* | **0** |
| Anoxic | Water | Control | 7.44 | 7.87 | *NA* | *NA* |
| Anoxic | Water | 50 | 7.23 | 8.33 | *NA* | *NA* |
| Anoxic | Water | 100 | 7.37 | 8.38 | *NA* | *NA* |
| Anoxic | Water | 200 | 8.11 | 7.97 | *NA* | *NA* |
| Anoxic | Water | 400 | 7.8 | 8.03 | *NA* | *NA* |
| Anoxic | Water | 600 | 7.99 | 8.24 | *NA* | *NA* |
| Anoxic | Water | 800 | 7.79 | 8.27 | *NA* | *NA* |
| Anoxic | Water | 1000 | 7.86 | 8.09 | *NA* | *NA* |
| Anoxic | Water | 2000 | 7.87 | 8.14 |  |  |
| Anoxic | Water | 4000 | 8.02 | 8.26 |  |  |
| Anoxic | Water | 8000 | 8.06 | 8.29 |  |  |

**Table S3:** Percentages of sediment organic carbon (Org C) and nitrogen (N) content and corresponding Org C:N in collected sediments used for batch incubations, and total nitrogen (TN) and dissolved organic carbon (DOC) from the manure used for batch and core incubations.

| Sediment type | % N | % Org C | Org C:N | TN mmol L^-1^ | DOC mmol L^-1^ |
| --- | --- | --- | --- | --- | --- |
| *Sediment used in manure-treated incubations* | | | | *NA* | |
| Oxic | 1.18 ± 0.06 | 17.41 ± 0.83 | 14.73 ± 0.47 |  |  |
| Anoxic | 1.19 ± 0.01 | 19.02 ± 0.39 | 15.93 ± 0.31 |  |  |
| *Sediment used in artificial fertilizer-treated incubations* | | | |  |  |
| Oxic | 0.86 ± 0.03 | 10.69 ± 0.20 | 12.40 ± 0.36 |  |  |
| Anoxic | 1.13 ± 0.04 | 17.15 ± 1.10 | 15.23 ± 0.49 |  |  |
| *Manure used for batch and core incubations* | | | |  |  |
| Manure extract | 2.65 ± 0.00 | 31.5 ± 0.28 | 11.9 ± 0.08 | 153.27 | 164.07 |

**Table S4:** Linear mixed model (LMM) and pairwise comparisons of repeated CH_4_ emissions between manure and oxygen treatments from core incubations.

| Full LMM model on log10 CH_4_ data | | | | | | |
| --- | --- | --- | --- | --- | --- | --- |
|  | **Sum Sq** | **Mean Sq** | **NumDF** | **DenDF** | **F value** | **P value** |
| Manure treatment | 35.07 | 11.69 | 3 | 23.35 | 20.94 | < 0.0001 |
| Time | 30.33 | 4.33 | 7 | 156.52 | 7.76 | < 0.0001 |
| Oxygen treatment | 49.09 | 49.09 | 1 | 23.39 | 87.95 | < 0.0001 |
| Manure treatment:Time | 70.86 | 3.37 | 21 | 156.43 | 6.05 | < 0.0001 |
| Manure treatment:Oxygen treatment | 7.81 | 2.61 | 3 | 23.35 | 4.67 | 0.01 |
| Days:Oxygen treatment | 4.18 | 0.60 | 7 | 156.52 | 1.07 | 0.39 |
| Manure treatment:Days:Oxygen treatment | 31.16 | 1.48 | 21 | 156.43 | 2.66 | 0.0003 |
|  |  |  |  |  |  |  |
| Tukey post hoc test | | | | | |  |
| contrast | **estimate** | **SE** | **df** | **t.ratio** | **p.value** |  |
| Control Anoxic - Low Anoxic | -0.26 | 0.42 | 22.70 | -0.62 | 0.998 |  |
| Control Anoxic - Mid Anoxic | -1.05 | 0.42 | 22.90 | -2.47 | 0.259 |  |
| Control Anoxic - High Anoxic | -1.38 | 0.42 | 22.90 | -3.24 | 0.059 |  |
| Control Anoxic - Control Oxic | 2.76 | 0.46 | 24.10 | 5.95 | 0.0001 |  |
| Control Anoxic - Low Oxic | 1.68 | 0.42 | 22.70 | 3.97 | 0.012 |  |
| Control Anoxic - Mid Oxic | 1.65 | 0.42 | 22.70 | 3.89 | 0.014 |  |
| Control Anoxic - High Oxic | -0.62 | 0.42 | 22.90 | -1.47 | 0.813 |  |
| Low Anoxic - Mid Anoxic | -0.78 | 0.42 | 22.90 | -1.84 | 0.600 |  |
| Low Anoxic - High Anoxic | -1.11 | 0.42 | 22.90 | -2.62 | 0.199 |  |
| Low Anoxic - Control Oxic | 3.02 | 0.46 | 24.10 | 6.52 | <.0001 |  |
| Low Anoxic - Low Oxic | 1.94 | 0.42 | 22.70 | 4.60 | 0.003 |  |
| Low Anoxic - Mid Oxic | 1.91 | 0.42 | 22.70 | 4.52 | 0.003 |  |
| Low Anoxic - High Oxic | -0.36 | 0.42 | 22.90 | -0.85 | 0.988 |  |
| Mid Anoxic - High Anoxic | -0.33 | 0.43 | 23.10 | -0.78 | 0.993 |  |
| Mid Anoxic - Control Oxic | 3.80 | 0.47 | 24.30 | 8.19 | <.0001 |  |
| Mid Anoxic - Low Oxic | 2.73 | 0.42 | 22.90 | 6.43 | <.0001 |  |
| Mid Anoxic - Mid Oxic | 2.69 | 0.42 | 22.90 | 6.35 | <.0001 |  |
| Mid Anoxic - High Oxic | 0.42 | 0.43 | 23.10 | 0.99 | 0.971 |  |
| High Anoxic - Control Oxic | 4.14 | 0.47 | 24.30 | 8.90 | <.0001 |  |
| High Anoxic - Low Oxic | 3.06 | 0.42 | 22.90 | 7.21 | <.0001 |  |
| High Anoxic - Mid Oxic | 3.02 | 0.42 | 22.90 | 7.13 | <.0001 |  |
| High Anoxic - High Oxic | 0.75 | 0.43 | 23.10 | 1.77 | 0.647 |  |
| Control Oxic - Low Oxic | -1.08 | 0.46 | 24.10 | -2.33 | 0.320 |  |
| Control Oxic - Mid Oxic | -1.11 | 0.46 | 24.10 | -2.40 | 0.286 |  |
| Control Oxic - High Oxic | -3.38 | 0.47 | 24.30 | -7.28 | <.0001 |  |
| Low Oxic - Mid Oxic | -0.03 | 0.42 | 22.70 | -0.08 | 1 |  |
| Low Oxic - High Oxic | -2.30 | 0.42 | 22.90 | -5.44 | 0.0004 |  |
| Mid Oxic - High Oxic | -2.27 | 0.42 | 22.90 | -5.36 | 0.0004 |  |

**Table S5:** Linear mixed model (LMM) and pairwise comparisons of repeated CO_2_ emissions between manure and oxygen treatments from core incubations.

| Full LMM model CO_2_ data | | | | | | | | | |
| --- | --- | --- | --- | --- | --- | --- | --- | --- | --- |
|  | | | **Sum Sq** | **Mean Sq** | | **NumDF** | **DenDF** | **F value** | **P value** |
| Manure treatment | | | 308.85 | 102.95 | | 3 | 23.20 | 10.61 | 0.0001 |
| Time | | | 769.59 | 109.94 | | 7 | 158.35 | 11.33 | < 0.0001 |
| Oxygen treatment | | | 45.71 | 45.71 | | 1 | 23.20 | 4.71 | 0.04 |
| Manure treatment:Days | | | 741.65 | 35.31 | | 21 | 158.33 | 3.64 | < 0.0001 |
| Manure treatment:Oxygen treatment | | | 65.94 | 21.97 | | 3 | 23.20 | 2.27 | 0.1 |
| Days:Oxygen treatment | | | 119.58 | 17.08 | | 7 | 158.35 | 1.76 | 0.09 |
| Manure treatment:Days:Oxygen treatment | | | 312.3 | 14.87 | | 21 | 158.33 | 1.53 | 0.07 |
|  |  |  |  | |  |  |  |  |  |
| Tukey post hoc test | | | | | | | | |  |
| contrast | | | **estimate** | **SE** | | **df** | **t.ratio** | **P value** |  |
| Control Anoxic - Low Anoxic | | | -2.31 | 1.68 | | 23.70 | -1.38 | 0.86 |  |
| Control Anoxic - Mid Anoxic | | | -4.13 | 1.66 | | 23.10 | -2.48 | 0.25 |  |
| Control Anoxic - High Anoxic | | | -8.78 | 1.66 | | 23.10 | -5.28 | 0.0005 |  |
| Control Anoxic - Control Oxic | | | -0.13 | 1.80 | | 23.00 | -0.07 | 1 |  |
| Control Anoxic - Low Oxic | | | -2.89 | 1.66 | | 23.10 | -1.73 | 0.67 |  |
| Control Anoxic - Mid Oxic | | | -0.21 | 1.66 | | 23.10 | -0.13 | 1 |  |
| Control Anoxic - High Oxic | | | -4.63 | 1.66 | | 23.10 | -2.78 | 0.15 |  |
| Low Anoxic - Mid Anoxic | | | -1.82 | 1.67 | | 23.50 | -1.09 | 0.95 |  |
| Low Anoxic - High Anoxic | | | -6.47 | 1.67 | | 23.50 | -3.87 | 0.015 |  |
| Low Anoxic - Control Oxic | | | 2.18 | 1.80 | | 23.40 | 1.21 | 0.92 |  |
| Low Anoxic - Low Oxic | | | -0.57 | 1.67 | | 23.50 | -0.34 | 1 |  |
| Low Anoxic - Mid Oxic | | | 2.10 | 1.67 | | 23.50 | 1.26 | 0.91 |  |
| Low Anoxic - High Oxic | | | -2.31 | 1.67 | | 23.50 | -1.38 | 0.86 |  |
| Mid Anoxic - High Anoxic | | | -4.65 | 1.66 | | 22.80 | -2.80 | 0.14 |  |
| Mid Anoxic - Control Oxic | | | 4.00 | 1.79 | | 22.80 | 2.23 | 0.37 |  |
| Mid Anoxic - Low Oxic | | | 1.24 | 1.66 | | 22.80 | 0.75 | 0.99 |  |
| Mid Anoxic - Mid Oxic | | | 3.92 | 1.66 | | 22.80 | 2.36 | 0.31 |  |
| Mid Anoxic - High Oxic | | | -0.50 | 1.66 | | 22.80 | -0.30 | 1 |  |
| High Anoxic - Control Oxic | | | 8.65 | 1.79 | | 22.80 | 4.83 | 0.0016 |  |
| High Anoxic - Low Oxic | | | 5.90 | 1.66 | | 22.80 | 3.55 | 0.031 |  |
| High Anoxic - Mid Oxic | | | 8.57 | 1.66 | | 22.80 | 5.17 | 0.0007 |  |
| High Anoxic - High Oxic | | | 4.16 | 1.66 | | 22.80 | 2.51 | 0.24 |  |
| Control Oxic - Low Oxic | | | -2.75 | 1.79 | | 22.80 | -1.54 | 0.78 |  |
| Control Oxic - Mid Oxic | | | -0.08 | 1.79 | | 22.80 | -0.04 | 1 |  |
| Control Oxic - High Oxic | | | -4.49 | 1.79 | | 22.80 | -2.51 | 0.24 |  |
| Low Oxic - Mid Oxic | | | 2.67 | 1.66 | | 22.80 | 1.61 | 0.74 |  |
| Low Oxic - High Oxic | | | -1.74 | 1.66 | | 22.80 | -1.05 | 0.96 |  |
| Mid Oxic - High Oxic | | | -4.41 | 1.66 | | 22.80 | -2.66 | 0.19 |  |

**Table S6:** Linear mixed model (LMM) and pairwise comparisons of repeated N_2_O emissions between manure and oxygen treatments from core incubations.

| Full LMM model N_2_O data | | | | | | | | | | | | | | | | | | | |
| --- | --- | --- | --- | --- | --- | --- | --- | --- | --- | --- | --- | --- | --- | --- | --- | --- | --- | --- | --- |
|  | | | **Sum Sq** | | | | **Mean Sq** | | | **NumDF** | | | **DenDF** | | **F value** | | | **P value** | |
| Manure treatment | | | 14.983 | | | | 4.994 | | | 3 | | | 226 | | 1.25 | | | 0.3 | |
| Time | | | 74.638 | | | | 74.638 | | | 1 | | | 226 | | 18.68 | | | < 0.0001 | |
| Oxygen treatment | | | 26.075 | | | | 26.075 | | | 1 | | | 226 | | 6.53 | | | 0.01 | |
| Manure treatment:Days | | | 25.974 | | | | 8.658 | | | 3 | | | 226 | | 2.17 | | | 0.09 | |
| Manure treatment:Oxygen treatment | | | 10.624 | | | | 3.541 | | | 3 | | | 226 | | 0.89 | | | 0.45 | |
| Days:Oxygen treatment | | | 1.244 | | | | 1.244 | | | 1 | | | 226 | | 0.31 | | | 0.58 | |
| Manure treatment:Days:Oxygen treatment | | | 10.25 | | | | 3.417 | | | 3 | | | 226 | | 0.86 | | | 0.47 | |
|  |  | | | | |  | | |  | | |  | |  | | |  | |  |
|  |  | | | | |  | | |  | | |  | |  | | |  | |  |
| Tukey post hoc test | | | | | | | | | | | | | | | |  |  |  |  |
| contrast | | **estimate** | | **SE** | **df** | | | **t.ratio** | | | **P value** | | | | |  |  |  |  |
| Control Anoxic - Low Anoxic | | -0.15 | | 0.52 | 24.80 | | | -0.30 | | | 1 | | | | |  |  |  |  |
| Control Anoxic - Mid Anoxic | | 0.07 | | 0.50 | 22.50 | | | 0.14 | | | 1 | | | | |  |  |  |  |
| Control Anoxic - High Anoxic | | -0.57 | | 0.50 | 22.50 | | | -1.13 | | | 0.94 | | | | |  |  |  |  |
| Control Anoxic - Control Oxic | | 1.30 | | 0.54 | 22.40 | | | 2.39 | | | 0.29 | | | | |  |  |  |  |
| Control Anoxic - Low Oxic | | 1.47 | | 0.51 | 23.20 | | | 2.88 | | | 0.12 | | | | |  |  |  |  |
| Control Anoxic - Mid Oxic | | 0.81 | | 0.51 | 23.20 | | | 1.59 | | | 0.75 | | | | |  |  |  |  |
| Control Anoxic - High Oxic | | 1.72 | | 0.50 | 22.50 | | | 3.42 | | | 0.041 | | | | |  |  |  |  |
| Low Anoxic - Mid Anoxic | | 0.22 | | 0.51 | 24.10 | | | 0.44 | | | 0.99 | | | | |  |  |  |  |
| Low Anoxic - High Anoxic | | -0.42 | | 0.51 | 24.10 | | | -0.81 | | | 0.99 | | | | |  |  |  |  |
| Low Anoxic - Control Oxic | | 1.45 | | 0.55 | 23.80 | | | 2.64 | | | 0.19 | | | | |  |  |  |  |
| Low Anoxic - Low Oxic | | 1.62 | | 0.52 | 24.80 | | | 3.13 | | | 0.072 | | | | |  |  |  |  |
| Low Anoxic - Mid Oxic | | 0.96 | | 0.52 | 24.80 | | | 1.87 | | | 0.59 | | | | |  |  |  |  |
| Low Anoxic - High Oxic | | 1.88 | | 0.51 | 24.10 | | | 3.66 | | | 0.023 | | | | |  |  |  |  |
| Mid Anoxic - High Anoxic | | -0.64 | | 0.50 | 21.80 | | | -1.28 | | | 0.90 | | | | |  |  |  |  |
| Mid Anoxic - Control Oxic | | 1.23 | | 0.54 | 21.80 | | | 2.28 | | | 0.35 | | | | |  |  |  |  |
| Mid Anoxic - Low Oxic | | 1.39 | | 0.50 | 22.50 | | | 2.77 | | | 0.15 | | | | |  |  |  |  |
| Mid Anoxic - Mid Oxic | | 0.74 | | 0.50 | 22.50 | | | 1.47 | | | 0.82 | | | | |  |  |  |  |
| Mid Anoxic - High Oxic | | 1.65 | | 0.50 | 21.80 | | | 3.31 | | | 0.053 | | | | |  |  |  |  |
| High Anoxic - Control Oxic | | 1.87 | | 0.54 | 21.80 | | | 3.46 | | | 0.039 | | | | |  |  |  |  |
| High Anoxic - Low Oxic | | 2.03 | | 0.50 | 22.50 | | | 4.04 | | | 0.01 | | | | |  |  |  |  |
| High Anoxic - Mid Oxic | | 1.38 | | 0.50 | 22.50 | | | 2.74 | | | 0.16 | | | | |  |  |  |  |
| High Anoxic - High Oxic | | 2.29 | | 0.50 | 21.80 | | | 4.59 | | | 0.0031 | | | | |  |  |  |  |
| Control Oxic - Low Oxic | | 0.17 | | 0.54 | 22.40 | | | 0.30 | | | 1 | | | | |  |  |  |  |
| Control Oxic - Mid Oxic | | -0.49 | | 0.54 | 22.40 | | | -0.90 | | | 0.98 | | | | |  |  |  |  |
| Control Oxic - High Oxic | | 0.42 | | 0.54 | 21.80 | | | 0.79 | | | 0.99 | | | | |  |  |  |  |
| Low Oxic - Mid Oxic | | -0.66 | | 0.51 | 23.20 | | | -1.29 | | | 0.9 | | | | |  |  |  |  |
| Low Oxic - High Oxic | | 0.26 | | 0.50 | 22.50 | | | 0.51 | | | 0.99 | | | | |  |  |  |  |
| Mid Oxic - High Oxic | | 0.91 | | 0.50 | 22.50 | | | 1.81 | | | 0.62 | | | | |  |  |  |  |

**Table S7:** Summary of the GAM relating manure dose to GHG emissions. CO_2_-equivalent emissions were modeled as a function of manure dose using a GAM with Gaussian errors and identity link. Gas type (CO_2_, CH_4_, N_2_O) and treatment (Oxic vs. Anoxic) were included as parametric factors, and gas- and treatment-specific smooth terms were fitted for manure dose using thin plate regression splines (k = 4). Model parameters were estimated using restricted maximum likelihood (REML).

| Model performance | | | | |
| --- | --- | --- | --- | --- |
|  | **Adj. r^2^** | **n** | **Explained deviance** | **REML** |
| Full model | 0.81 | 93 | 82.7% | 222.18 |

| Parametric terms | | | | |
| --- | --- | --- | --- | --- |
|  | **Estimate** | **SE** | **t-value** | **p-value** |
| Intercept (N_2_O, Oxic) | -1.39 | 0.60 | -2.31 | 0.023 |
| Gas (CH_4_) | 7.72 | 0.73 | 10.53 | <0.0001 |
| Gas (CO_2_) | 9.65 | 0.73 | 13.16 | <0.0001 |
| Treatment (Anoxic) | 3.25 | 0.60 | 5.42 | <0.0001 |

| Smooth terms | | | |
| --- | --- | --- | --- |
|  | **edf** | **F** | **p-value** |
| Manure dose – N_2_O (Oxic) | 1.00 | 0.00 | 0.978 |
| Manure dose – CH_4_ (Oxic) | 1.00 | 50.18 | <0.0001 |
| Manure dose – CO_2_ (Oxic) | 1.00 | 4.00 | 0.049 |
| Manure dose – N_2_O (Anoxic) | 1.00 | 0.02 | 0.89 |
| Manure dose – CH_4_ (Anoxic) | 1.91 | 47.69 | <0.0001 |
| Manure dose – CO_2_ (Anoxic) | 1.52 | 8.79 | <0.001 |

**Table S8:** Average CO_2_, CH_4_ and N_2_O emissions from core incubations for each oxygen and manure treatment throughout a 27-day incubation period. Emissions are given in absolute units and as CO_2_-equivalent.

|  |  | CO_2_ | | CH_4_ | | N_2_O | |
| --- | --- | --- | --- | --- | --- | --- | --- |
| Oxygen treatment | **Manure treatment** | g CO_2_ m^-2^ d^-1^ | g CO_2_-eq m^-2^ d^-1^ | g CH_4_ m^-2^ d^-1^ | g CO_2_-eq m^-2^ d^-1^ | mg N_2_O m^-2^ d^-1^ | g CO_2_-eq m^-2^ d^-1^ |
| Oxic | Control | 7.05±1.04 | 7.05±1.04 | 0.013±0.005 | 0.35±0.12 | 0.28±0.59 | 0.08±0.16 |
| Oxic | Low | 9.8±2.96 | 9.8±2.96 | 0.05±0.05 | 1.4±1.3 | 0.09±0.67 | 0.02±0.18 |
| Oxic | Mid | 7.12±1.89 | 7.12±1.89 | 0.04±0.02 | 1.2±0.48 | 0.74±0.23 | 0.2±0.06 |
| Oxic | High | 11.54±4.78 | 11.54±4.78 | 0.49±0.2 | 13.14±5.4 | 0.14±0.32 | -0.04±0.09 |
| Anoxic | Control | 6.81±0.78 | 6.81±0.78 | 0.17±0.1 | 4.51±2.67 | 1.57±0.41 | 0.43±0.11 |
| Anoxic | Low | 9.46±1.94 | 9.46±1.94 | 0.22±0.14 | 5.9±3.8 | 1.74±0.31 | 0.48±0.09 |
| Anoxic | Mid | 11.05±1.21 | 11.05±1.21 | 0.46±0.1 | 12.4±2.7 | 1.56±0.51 | 0.43±0.14 |
| Anoxic | High | 15.7±0.66 | 15.7±0.66 | 0.84±0.24 | 22.8±6.4 | 2.23±0.54 | 0.61±0.15 |
